# Hyperthyroidism with Turner Syndrome

**DOI:** 10.1186/1687-9856-2013-S1-P140

**Published:** 2013-10-03

**Authors:** Ratna Dewi Artati, Satriono

**Affiliations:** 1 Department of Child Health, Medical School, University of Hasanuddin, Makassar, Indonesia

**Keywords:** hyperthyroidism, short stature, autoimmune, Turner syndrome

## Background

Thyroid dysfunction is more frequently encountered in girls with Turner syndrome (TS). Moreover, patients with both Graves's disease and the TS have been reported only rarely. Recent evidence indicates that Graves's disease is also a disorder of cell mediated immunity.

## Objective

To report a case of 10-year and 8-month-old girl with hyperthyroidism and TS.

## Case

A 10-year and 8-month-old girl presented with short stature (body weight 22 kg, body height 116 cm). On physical examination there were bilateral exophthalmus, enlargement of thyroid grade IA, widely spaced nipples, small finger nails. Laboratory findings showed elevated free thyroxin (FT4), decreased sensitive thyroid stimulating hormone (TSHs) and positive anti thyroglobuline. Ultrasonography (USG) thyroid gland revealed a diffuse enlargement. The ophthalmology examination result was exophthalmus with normal visus. No abnormality on the chest X-Ray and electrocardiography. Bone age was equal to a 8-year and 10-month-old girl. Chromosom analysis result : Mosaik 45, X / 46, Xr(X). Treatment with carbimazole have provided some improvement.

## Conclusion

The diagnosis was established based on anamnesis, physical examination, laboratory findings and chromosom analysis. Prognosis for this case is undefined.

